# Species differences in pancreatic binding of DO3A-VS-Cys^40^-Exendin4

**DOI:** 10.1007/s00592-017-1046-2

**Published:** 2017-09-11

**Authors:** Olof Eriksson, Ulrika Rosenström, Ram K. Selvaraju, Barbro Eriksson, Irina Velikyan

**Affiliations:** 1 Department of Medicinal Chemistry, Uppsala University, Dag Hammarskjölds väg 14C, 3tr, SE-751 83 Uppsala, Sweden; 2 Department of Medical Sciences, Uppsala University, Uppsala, Sweden

**Keywords:** Beta cell imaging, Exendin4, Positron emission tomography, Beta cell mass, Animal models, GLP-1R

## Abstract

**Aims:**

Radiolabeled Exendin-4 has been proposed as suitable imaging marker for pancreatic beta cell mass quantification mediated by Glucagon-like peptide-1 receptor (GLP-1R). However, noticeable species variations in basal pancreatic uptake as well as uptake reduction degree due to selective beta cell ablation were observed.

**Methods:**

In vitro and ex vivo autoradiography studies of pancreas were performed using [^177^Lu]Lu-DO3A-VS-Cys^40^-Exendin4, in order to investigate the mechanism of uptake as well as the islet uptake contrast in mouse, rat, pig, and non-human primate. The autoradiography results were compared to the in vivo pancreatic uptake as assessed by [^68^Ga]Ga-DO3A-VS-Cys^40^-Exendin4 Positron Emission Tomography (PET) in the same species. In vitro, ex vivo, and in vivo data formed the basis for calculating the theoretical in vivo contribution of each pancreatic compartment.

**Results:**

[^177^Lu]Lu-DO3A-VS-Cys^40^-Exendin4 displayed the highest islet-to-exocrine pancreas ratio (IPR) in rat (IPR 45) followed by non-human primate and mouse at similar levels (IPR approximately 5) while pigs exhibited negligible IPR (1.1). In vivo pancreas uptake was mainly GLP-1R mediated in all species, but the magnitude of uptake under basal physiology varied significantly in decreasing order: non-human primate, mouse, pig, and rat. The theoretical calculation of islet contribution to the total pancreatic PET signal predicted the in vivo observation of differences in pancreatic uptake of [^68^Ga]Ga-DO3A-VS-Cys^40^-Exendin4.

**Conclusions:**

IPR as well as the exocrine GLP-1R density is the main determinants of the species variability in pancreatic uptake. Thus, the IPR in human is an important factor for assessing the potential of GLP-1R as an imaging biomarker for pancreatic beta cells.

## Introduction

In vivo imaging of the endocrine pancreas requires labeled probes with high affinity for a molecular target expressed specifically in the beta cells [1, 2]. Glucagonlike peptide-1 receptor (GLP-1R) is an important pharmaceutical target in the treatment of type 2 diabetes (T2D) and has been proposed as a surrogate imaging biomarker for beta cell mass [1]. Exendin-4, a peptidomimetic of the endogenous ligand GLP-1 is resistant to both dipeptidyl peptidase IV and neural endopeptidase and therefore a suitable peptide for imaging purposes. Several radiolabeled analogues of Exendin-4 have been evaluated for critical parameters such as affinity to GLP-1R and beta cell selectivity in vivo. Especially, rats display a strong contrast between beta cell uptake compared to that of the exocrine pancreatic background uptake (islet-to-exocrine pancreas ratio, IPR). Subsequently, it has been feasible to detect decreased beta cell mass in rat models of diabetes [3, 4].

Accumulating studies during recent years have demonstrated marked differences in the pancreatic uptake level of radiolabeled Exendin-4 among various species with the most pronounced discrepancy between rats and such species as mouse [5, 6], pig [7], and non-human primate [3]. Especially, the exocrine binding seems to vary as beta cell ablated T1D models display large difference in the residual, non-beta cell mediated uptake of radiolabeled Exendin-4 with least uptake in T1D rat and increasing in magnitude in T1D models of mouse [4], pig [7], and non-human primate [8].

The apparent pancreatic uptake as measured by Positron Emission Tomography (PET) reflects the collective function of all pancreatic compartments: the endocrine compartment comprising mainly the islets of Langerhans (1–3% of the pancreas volume), the exocrine compartment (80–90% of the pancreas volume) as well as blood vessels and ducts. In order to improve the understanding of the mechanism of uptake of DO3A-VS-Cys^40^-Exendin4 in the different pancreatic compartments, we performed in vitro and ex vivo autoradiography studies using [^177^Lu]Lu/[^68^Ga]Ga-DO3A-VS-Cys^40^-Exendin4, in comparison with the in vivo pancreatic uptake as assessed by [^68^Ga]Ga-DO3A-VS-Cys^40^-Exendin4/PET.

## Methods and materials

### Radiochemistry

[^177^Lu]Lu-DO3A-VS- Cys^40^-Exendin-4 [9] and [^68^Ga]Ga-DO3A-VS- Cys^40^-Exendin4 [2, 10] were synthesized as described previously.

### In vitro autoradiography

Pancreatic tissue was collected from non-diabetic rat, pigs, and non-human primate. Explanted INS-1 (rat beta cell origin) xenograft tumors were collected from immunodeficient mice [11]. Biopsies from resected human pancreatic insulinoma (*n* = 3) were obtained by the section for Endocrine Oncology, Uppsala University Hospital and its use was approved by EPN 2013/460 according to the Declaration of Helsinki. All subjects gave their informed consent.

All biopsies were stored at −80 °C, embedded in CryoFix and processed into 20 µm slices. [^177^Lu]Lu-DO3A-VS-Cys^40^-Exendin4 corresponding to approximately 0.05–30 nM peptide mass in a total volume of 150 mL 50 mM TRIS was added followed by incubation for 60 min at room temperature. Non-displaceable binding was assessed by adding 200 nM of unlabeled Exendin-4 or GLP-1.

After incubation, the tissue sections were washed 3 times for 4 min in 150 ml 50 mM TRIS at 4 °C to remove unbound [^177^Lu]Lu-DO3A-VS-Cys^40^-Exendin4 and then dried at 37 °C for 10 min. Finally, the sections were exposed against a phosphorimager screen together with a reference droplets of known activity of [^177^Lu]Lu-DO3A-VS-Cys^40^-Exendin4 overnight and then scanned using a Cyclone Plus Phosphorimager (Perkin Elmer) at 600 dpi. [^68^Ga]Ga-DO3A-VS- Cys^40^-Exendin4 was used as radioligand the frozen section autoradiography of human insulinoma sections.

The autoradiograms were analyzed using ImageJ (NIH). Affinity (expressed as the dissociation constant K_d_) and receptor density (B_max_) were determined by nonlinear regression of the receptor bound tracer amount as function of the added tracer concentration, using GraphPad Prism 6 (San Diego, CA, USA).

### Ex vivo autoradiography of pancreas

All applicable international, national, and/or institutional guidelines for the care and use of animals were followed. All procedures performed in studies involving animals were in accordance with the ethical standards of the institution or practice at which the studies were conducted.

[^177^Lu]Lu-DO3A-VS-Cys^40^-Exendin4 was administered intravenously (IV) in sedated animals according to Table 1. The administered peptide dose was maintained low (<0.8 µg/kg) to minimize mass effects and inadvertent partial receptor saturation by the tracer itself. After 60 min, pancreatic biopsies were immediately collected postmortem and frozen in liquid nitrogen. The biopsies were embedded in CryoFix, processed into 20 µm slices and immediately placed on a phosphorimager screen and exposed overnight. The phosphorimager screen was developed in a Cyclone phosphorimager (PerkinElmer) and the raw data image stored digitally. Images were analyzed using ImageJ (NIH). Fluorescent insulin staining was performed as described previously [12].

**Table 1:** Administration information regarding peptide dose for the ex vivo autoradiography studies in mouse, rat, pig, and non-human primate

Species	*N*	Peptide dose (µg/kg)	Time (post-injection) (min)
Mouse	8	0.8	60
Rat	2	0.1	60
Pig	2	0.1	60
NHP	2	0.05	60

### Retrospective evaluation and comparison of in vivo species differences in total GLP-1 receptor density

Pancreatic uptake of [^68^Ga]Ga-DO3A-VS-Cys^40^-Exendin4 in different species was summarized based on our previous studies: mouse (*n* = 6, nu/nu Balb/c) [9], rat (*n* = 5, Lewis) [2], pig (*n* = 4) [6], and non-human primate (*n* = 3, cynomolgus) [2].

The pancreatic radioactive uptake (Bq/cc) was expressed as standardized uptake values (SUVs) by normalizing by the administered radioactive dose (Bq) and subject weight (g). The normalization allows the comparison of SUVs between individuals and species.

In order to reduce confounding factors such as differences in vascular contribution and washout, the pancreatic SUVs are reported at 60 min (80 min for mouse) following IV administration of [^68^Ga]Ga-DO3A-VS-Cys^40^-Exendin4, either measured by organ distribution studies (mouse and rat) or PET imaging (pig and non-human primate). The total amount of the injected peptide per kg body weight of animals was maintained as low and similar as possible in order to minimize variability due to potential mass effects, while still providing the amount of the injected radioactivity dose sufficient for statistically viable counts for adequate signal registration (Table 2). The mice were given in the range of 0.5 MBq [^68^Ga]Ga-DO3A-VS-Cys^40^-Exendin4, which was judged as the lowest possible radioactive dose still being detectable by well counter in the organ distribution study. Despite optimized specific radioactivity for the ^68^Ga radiolabeling, this corresponded to 0.05 µg DO3A-VS-Cys^40^-Exendin4. This gives a lowest feasible baseline mass dose of 2.5 µg/kg due to the small size of the mice in the study (in the range of 20 g). As a comparison, the same mass dose in a 300 g rat corresponds to just above 0.1 µg/kg.

**Table 2:** Baseline dose of [^68^Ga]GaDO3A-VS-Cys^40^-Exendin4 and competing dose of Exendin-4 given to different species for the in vivo biodistribution studies

Species	Baseline peptide dose (µg/kg)	Competing peptide dose (µg/kg)	Time of PET imaging (post-injection) (min)
Mouse	2.5	100	80
Rat	0.1	100	60
Pig	0.1	3	60
NHP	0.05	20	60

The pancreatic SUV following co-injection of a pharmacological dose of DO3A-VS-Cys^40^-Exendin4 peptide is also reported in order to compare the off-target binding between species.

### Theoretical in vivo contribution of each pancreatic compartment based on in vitro, ex vivo, and in vivo data

In order to improve the understanding of the mechanism for the observed species differences of the in vivo uptake of [^68^Ga]Ga-DO3A-VS-Cys^40^-Exendin4, the theoretical contribution of the GLP-1R mediated binding in each pancreatic compartment as well as the off-target binding can be calculated.

The total pancreatic SUV (SUV_TOT_) was obtained from in vivo data (Fig. 3). In vivo off-target binding (SUV_OFFTARGET_) was assumed to be 10% in all species, based on the observation that up to 90% of the baseline [^68^Ga]Ga-DO3A-VS-Cys^40^-Exendin4 can be abolished by pre-treating with high doses unlabeled Exendin4, given enough time for washout (>90 min) [2]. The SUV mediated by GLP-1R (SUV_GLP1R_) can then be calculated as SUV_TOT_ − SUV_TOT_ × 10%/100.

The contribution of the islets (CONTR_ISLETS_) will be the product of the percentage of islets in pancreas and the IPR (as determined by ex vivo autoradiography in Fig. 2). The contribution of the exocrine tissue will be the product of only the percentage of exocrine tissue in pancreas (CONTR_EXOCRINE_).

The portion of the pancreas SUV mediated by GLP-1R being contributed by the islets (SUV_GLP1R, ISLETS_) can then be calculated by multiplying SUV_GLP1R_ with CONTR_ISLETS_/(CONTR_ISLETS_ × CONTR_EXOCRINE_) × 100. The portion of the pancreas SUV mediated by GLP-1R being contributed by the exocrine tissue (SUV_GLP1R, EXOCRINE_) is calculated in similar fashion.

Finally, given the calculated values for SUV_GLP1R, ISLETS_, SUV_GLP1R, EXOCRINE_, SUV_OFFTARGET_, the percentual contribution for each compartment to observed SUV_TOT_ can be determined.

### Statistics

Data on group level are reported as mean ± SD in all figures. Statistical analysis was performed and figures generated using GraphPad Prism 5 (GraphPad, La Jolla, CA, USA). Testing for normal distribution was performed by the D’Agostino & Pearson omnibus normality test. Differences between multiple groups were assessed by non-parametric Kruskal–Wallis one-way ANOVA with Dunns’s post hoc multiple comparisons test using a significance level of *p* < 0.05.

## Results

### Affinity and GLP-1R density in different species as assessed by [^177^Lu]Lu-DO3A-VS-Cys^40^-Exendin4 in vitro autoradiography

**Fig. 1:**
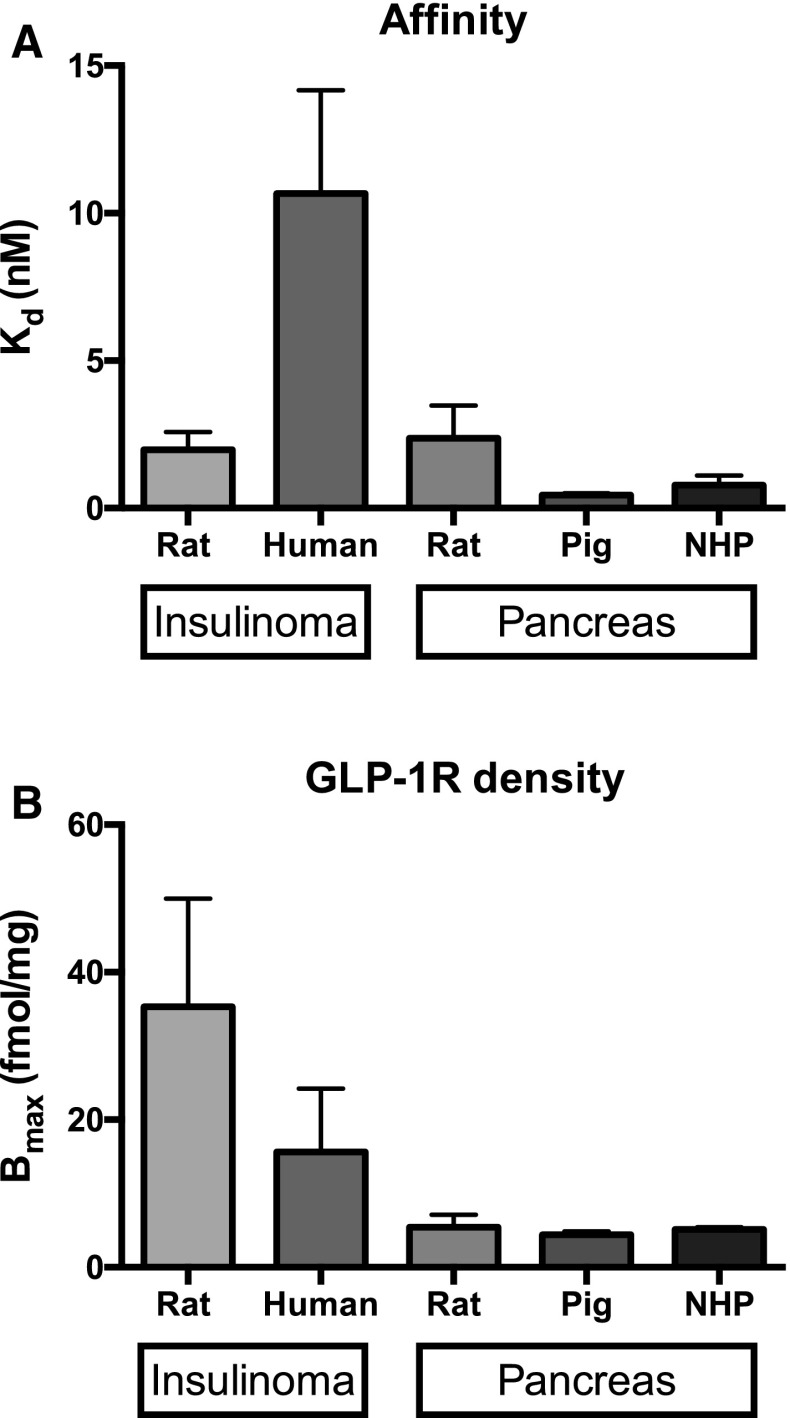
Affinity (**a**) and GLP-1R receptor density (**b**) in insulinoma and pancreas from several species as assessed by saturation binding experiments with radiolabeled DO3A-VS-Cys^40^-Exendin-4. Error bars represent standard deviations

Rat displayed nanomolar affinity in sections from either insulinoma (2.0 ± 1.0 nM) or pancreas (2.4 ± 1.1 nM) (Fig. 1a). DO3A-VS-Cys^40^-Exendin4 displayed lower affinity in human insulinoma (10.7 ± 3.5 nM). Pig (0.4 ± 0.1 nM) and to a lesser extent non-human primate (0.8 ± 0.3 nM) exhibited a tendency for higher affinity of DO3A-VS-Cys^40^-Exendin4 toward GLP-1R as compared to rat. Insulinoma exhibited higher GLP-1R density than normal pancreatic sections as expected (Fig. 1b).

### Ex vivo autoradiography of [^177^Lu]Lu-DO3A-VS-Cys^40^-Exendin4 binding in the pancreas

**Fig. 2:**
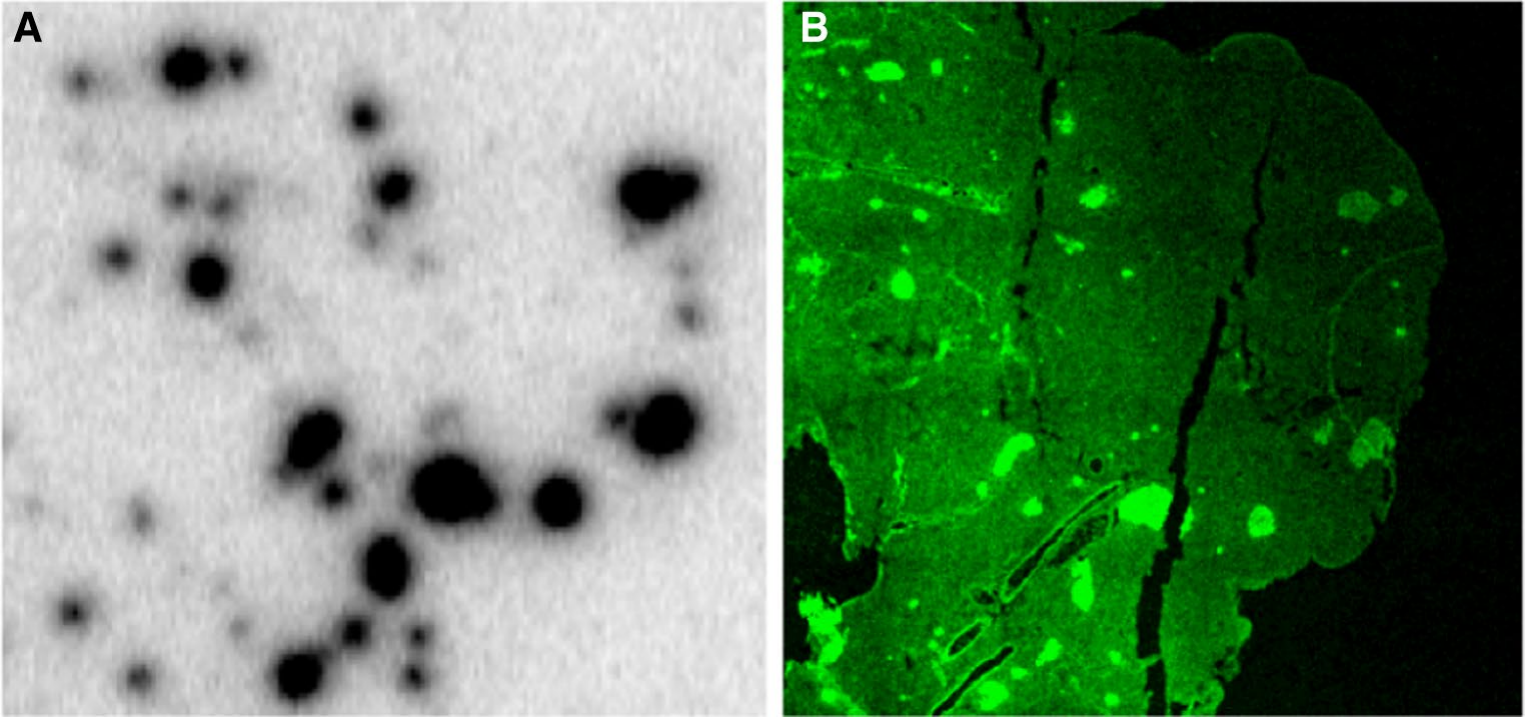
Example of co-localization between insulin staining (**a**) and [^177^Lu]Lu-DO3A-VS-Cys^40^-Exendin4 (**b**) in rat pancreas as assessed by ex vivo autoradiography

**Fig. 3:**
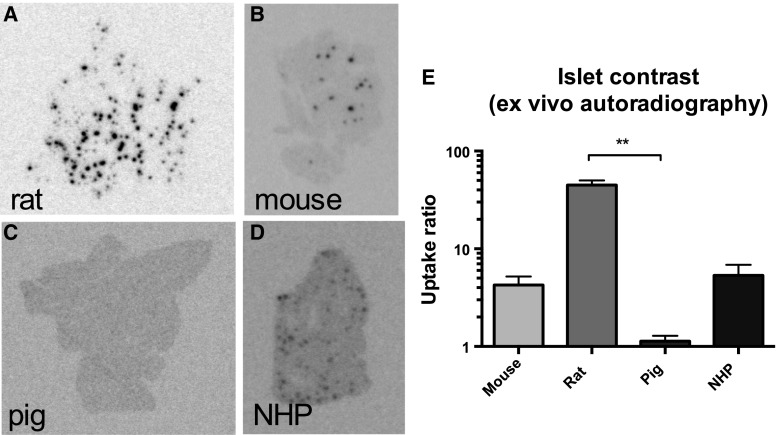
Ex vivo autoradiograms of pancreas in vivo distribution of [^177^Lu]Lu-DO3A-VS-Cys^40^-Exendin4 in rat (**a**), mouse (**b**), pig (**c**), and non-human primate (**d**). The islet contrast (**e**) defined as the islets-to-exocrine pancreas (IPR) ratio was highly dependent on the species, mainly reflecting the difference in background binding. Error bars represent standard deviations. **denote *p* < 0.01, ***denote *p* < 0.001 and ****denote *p* < 0.0001

The pancreatic ex vivo autoradiograms show the actual within-tissue in vivo binding pattern of DO3A-VS-Cys^40^-Exendin4 in pancreas. Rat sections displayed clearly defined islets (co-localization between [^177^Lu]Lu-DO3A-VS-Cys^40^-Exendin4 and insulin is shown in Fig. 2) in combination with low background binding in the exocrine pancreas (islet contrast 45 ± 5, Fig. 3a, e). Islets in mouse (islet contrast 4.3 ± 1.0, Fig. 3b) and non-human primate (islet contrast 5.3 ± 1.5, Fig. 3d) were identifiable above the background, but with a magnitude lower islet contrast than in rat pancreas. Pig islets on the other hand showed negligible contrast above the exocrine background (islet contrast 1.1 ± 0.2, Fig. 3c).

### Retrospective evaluation and comparison of in vivo species differences in total GLP-1 receptor density

**Fig. 4:**
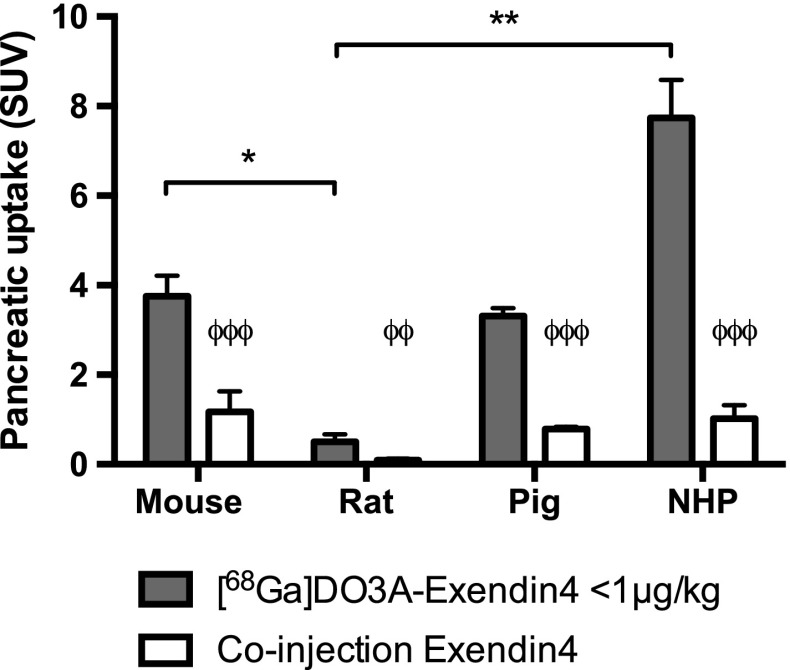
In vivo uptake (SUV) of [^68^Ga]Ga-DO3A-VS-Cys^40^-Exendin4 in pancreas of different species. *Solid bars* denote uptake at baseline doses, while *blue bars* with *red stripes* *white bars* denote uptake after co-injection of unlabeled DO3A-VS-Cys^40^-Exendin4 i.e., non-specific binding. ϕ denote difference in binding of [^68^Ga]Ga-DO3A-VS-Cys^40^-Exendin4 from baseline compared to co-injection of unlabeled peptide. *Star* denotes difference in pancreatic uptake between species at baseline conditions. *Error bars* represent standard deviations. *denote *p* < 0.05 and **denote *p* < 0.01 and ***denote *p* < 0.001 (color figure online)

Pancreatic uptake of [^68^Ga]Ga-DO3A-VS-Cys^40^-Exendin4 exhibited a striking variation among the species (Fig. 4). Non-human primate (SUV 7.7 ± 0.9) pancreatic uptake was more than a magnitude higher than in rat (SUV 0.51 ± 0.16). Mouse and pig displayed a pancreatic uptake (SUV 3.8 ± 0.5 and SUV 3.3 ± 0.2, respectively) lower than in non-human primate.

The uptake of [^68^Ga]Ga-DO3A-VS-Cys^40^-Exendin4 could be abolished by co-injection of an excess of DO3A-VS-Cys^40^-Exendin4 in all species with rat displaying the lowest off-target binding in vivo.

### Theoretical in vivo contribution of each pancreatic compartment based on in vitro, ex vivo, and in vivo data

**Fig. 5:**
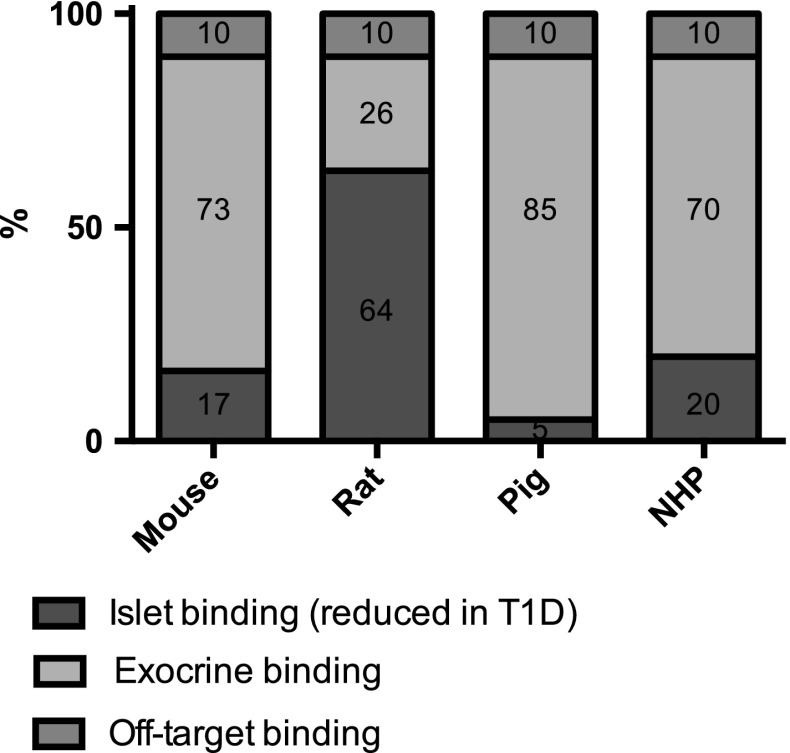
Theoretical binding pattern of [^68^Ga]Ga-DO3A-VS-Cys^40^-Exendin4 in pancreas in vivo, based on the species dependent islet contrast observed by ex vivo autoradiography. Each entire bar represents 100% of the uptake in pancreas in respective species, and each colored field is proportional in area to its predicted contribution to the total in vivo PET signal. The contribution of GLP-1R mediated binding in islets is indicated in *blue*. The predicted GLP-1R mediated binding in exocrine pancreas is indicated in *bright red*. Off-target binding, i.e., non-specific binding not mediated by GLP-1R throughout the pancreas, is indicated in yellow (color figure online)

The theoretical signal contribution of [^68^Ga]Ga-DO3A-VS-Cys^40^-Exendin4 binding in islets, in exocrine pancreas as well as the off-target binding is presented in Fig. 5. Due to the high IPR, rat islets (64%) are predicted to have higher percentual contribution relative to the islets in mouse (17%), NHP (20%) and especially the pig (5%). Conversely, the sum of the exocrine contribution and the off-target binding (which will be unaffected by acute beta cell ablation) is highest in pig (85 + 10 = 95%) followed in falling order by mouse (83%), NHP (80%) and rat (36%).

## Discussion

We present notable species variability in the pancreatic uptake and islet contrast of radiolabeled DO3A-VS-Cys^40^-Exendin4. These findings have important impact on the applications of GLP-1R imaging in vivo, for example as a beta cell marker, and interpretation of the imaging results.

The pancreatic uptake of DO3A-VS-Cys^40^-Exendin4 at basal non-diabetic physiology was species dependent as demonstrated in Fig. 4. Similar species differences between rats and mice have previously been reported for other radiolabeled Exendin4 analogues [4]. The in vivo uptake of a tracer in a target tissue is dependent on many factors, notably the affinity between the tracer and target, target expression density, tissue perfusion (tracer delivery and washout), mass effect, tracer metabolism, and off-target binding. In our studies, the mass effect was controlled by administration of low total amount of the peptide and similar peptide dose per kg animal weight. The early tracer delivery differences were minimized by measuring the in vivo uptake after >60 min wherein the uptake achieved the plateau. Off-target binding appears to vary somewhat in absolute levels (Fig. 4), but is fairly constant as a percentage of the baseline levels (10–30%) and there are no expected mechanisms to suspect species variability in off-target binding. The affinity is nanomolar with small inter-species variability except for human insulinoma GLP-1R. Tracer metabolism of [^68^Ga]Ga-DO3A-VS-Cys^40^-Exendin4 was not measured; however, generally rodents exhibit more rapid excretion of radiolabeled peptides compared to pig and non-human primate. Rapid metabolism could partially explain the relatively low pancreas binding of [^68^Ga]Ga-DO3A-VS-Cys^40^-Exendin4 observed binding in rat due to excretion. However, the similarly rapid metabolism in mouse does not result in low pancreas binding–on the contrary, the mouse displays relatively high binding of [^68^Ga]Ga-DO3A-VS-Cys^40^-Exendin4 in pancreas. Thus, it is instead reasonable that observed differences in [^68^Ga]Ga-DO3A-VS-Cys^40^-Exendin4 uptake reflect physiologically meaningful differences in GLP-1R mediated binding in the pancreas.

In order to assess the internal distribution pattern of radiolabeled DO3A-VS-Cys^40^-Exendin4 in different pancreatic compartments in vivo, we performed ex vivo autoradiography of pancreas sections obtained postmortem from mouse, rat, pig, and NHP administered with both [^177^Lu]LuDO3A-VS-Cys40-Exendin4 and [^68^Ga]Ga-DO3A-VS-Cys^40^-Exendin4. The ex vivo autoradiography analysis reveals unambiguous differences in the IPR. IPR was by far the highest in rats followed by non-human primates and mouse. Pigs fall in a separate category with negligible islet contrast.

Interestingly, there was no close correlation between the islet contrast and the magnitude of the total pancreatic uptake in any of the species. This observation, combined with the low off-target binding of [^68^Ga]Ga-DO3A-VS-Cys^40^-Exendin4 in all species, suggests that GLP-1R expression not only by islets but also by the exocrine compartments has a strong impact on [^68^Ga]Ga-DO3A-VS-Cys^40^-Exendin4 uptake.

This notion is further compounded by previous observations that beta cell deficient animal models of T1D have demonstrated different magnitudes of decrease in uptake of radiolabeled Exendin-4. Streptozotocin (STZ) treated rats (with relatively low pancreatic uptake also at basal non-diabetic physiology) exhibit a drastic decrease (>80%) in pancreatic binding [3] while, for example, STZ-treated pigs (with confirmed total beta cell ablation) show a completely unchanged strong pancreatic uptake [7]. The exact identity of the exocrine receptors to which bind DO3A-VS-Cys^40^-Exendin4, and their possible function, is currency unknown.

PET examinations measure the uptake of entire pancreas and have intrinsically insufficient spatial resolution to resolve individual islets. Therefore, the pancreatic in vivo signal will be formed by an amalgam of GLP-1R mediated binding in both the islet and exocrine pancreas, together with the off-target binding in the entire pancreas.

The calculations predicted that rat pancreas have the largest contribution from islets. This means that ablation of the islets from the rat pancreas by, for example, T1D induction could generate a reduction in [^68^Ga]Ga-DO3A-VS-Cys^40^-Exendin4 uptake by up to 64% which correlates well with a very high islet contrast of approximately 50. The islets in mouse and non-human primate on the other hand exhibited smaller signal contribution to the total pancreatic radioactive uptake compared to rat, despite a respectable islet contrast of approximately 5. Finally, pig islet uptake contributes only 5% to the total pancreas PET signal due to the poor IPR.

These theoretical calculations predicted the observed decrease in pancreatic binding due to induced beta cell ablation or development of T1D with reasonable accuracy (linear regression *R* ^2^ = 0.86): approximately, 80% in rat, 30% in mouse [6], and negligible decrease in pig [7] and non-human primate [8]. This demonstrates how the main ingoing parameter for the calculations, i.e., IPR, has strong quantifiable impact on the possibility of measuring decrease in beta cell mass.

## Conclusion

We present in vitro and ex vivo autoradiography data on the binding mechanism and distribution of [^68^Ga]GaDO3A-VS-Cys^40^-Exendin4 and [^177^Lu]LuDO3A-VS-Cys40-Exendin4 in pancreas. The results indicate that the islet contrast and the background GLP-1R density in the exocrine pancreas are the main determinant of the species variability in apparent pancreatic uptake. Thus, the islet contrast in human is an important factor for assessing its potential as an imaging biomarker for pancreatic beta cells.
